# The Cytotoxicity Mechanism of 6-Shogaol-Treated HeLa Human Cervical Cancer Cells Revealed by Label-Free Shotgun Proteomics and Bioinformatics Analysis

**DOI:** 10.1155/2012/278652

**Published:** 2012-11-11

**Authors:** Qun Liu, Yong-Bo Peng, Lian-Wen Qi, Xiao-Lan Cheng, Xiao-Jun Xu, Le-Le Liu, E-Hu Liu, Ping Li

**Affiliations:** State Key Laboratory of Natural Medicines, Department of Pharmacognosy, China Pharmaceutical University, Nanjing 210009, China

## Abstract

Cervical cancer is one of the most common cancers among women in the world. 6-Shogaol is a natural compound isolated from the rhizome of ginger (*Zingiber officinale*). In this paper, we demonstrated that 6-shogaol induced apoptosis and G2/M phase arrest in human cervical cancer HeLa cells. Endoplasmic reticulum stress and mitochondrial pathway were involved in 6-shogaol-mediated apoptosis. Proteomic analysis based on label-free strategy by liquid chromatography chip quadrupole time-of-flight mass spectrometry was subsequently proposed to identify, in a non-target-biased manner, the molecular changes in cellular proteins in response to 6-shogaol treatment. A total of 287 proteins were differentially expressed in response to 24 h treatment with 15 **μ**M 6-shogaol in HeLa cells. Significantly changed proteins were subjected to functional pathway analysis by multiple analyzing software. Ingenuity pathway analysis (IPA) suggested that 14-3-3 signaling is a predominant canonical pathway involved in networks which may be significantly associated with the process of apoptosis and G2/M cell cycle arrest induced by 6-shogaol. In conclusion, this work developed an unbiased protein analysis strategy by shotgun proteomics and bioinformatics analysis. Data observed provide a comprehensive analysis of the 6-shogaol-treated HeLa cell proteome and reveal protein alterations that are associated with its anticancer mechanism.

## 1. Introduction

Cervical cancer is the third most common female cancer in the world. Statistical data of 2008 show estimated 529,800 new diagnosed cervical cancer cases and 275,100 deaths among women worldwide [1]. Radiotherapy combined with chemotherapy is the dominant protocol to treat cervical cancer in clinical [2]. Because of the severe side effects, a lot of the patients cannot tolerant the chemotherapy drug [3]. In the light of the high prevalence of cervical cancer and refractoriness of malignant tumors to chemotherapy, it is urgent to have alternative concepts to the control and management of cancer. Natural dietary agents, especially spices and herbs, have attracted considerable attention due to their various properties in health promotion including suppression of cancers [4]. 

**Figure 1:**
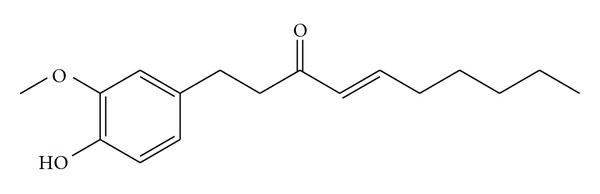
The chemical structure of 6-shogaol.

Ginger (*Zingiber officinale* Rosco, Zingiberaceae) has been used widely as a spicy or flavoring agent, and as a medicinal herb in traditional medicine. It has been utilized in oriental medicine for the treatment of ameliorate symptoms such as nausea, gastrointestinal discomforts, headache and cold [5]. The pungent phenolic constituents derived from ginger are believed to possess many interesting pharmacological and physiological activities. 6-Shogaol [(E)-1-(4-hydroxy-3-methoxyphenyl)-dec-4-en-3-one] (Figure 1), a major biologically active compound of ginger, was reported to exhibit anticancer efficacy. Multiple targets and pathways are found to be associated with 6-shogaol mediated cancer cell death, such as extracellular signal-regulate kinase 1/2, c-Jun N-terminal Kinase 1/2, p38 mitogen-activated protein kinase, phosphatidylinositol 3-kinase/Akt and cell cycle checkpoint proteins cdk1, cyclin B and cdc25C [6, 7]. However, neither the effect of 6-shogaol apoptosis induction nor the relationships between 6-shogaol lethality and cell signaling cascades has been examined in depth in cervical cancer cells. Simple cytotoxicity assays and conventional cytotoxic target detection can elicit the preliminary mechanism of the compound partly, but the approach cannot elucidate the global molecular targets. Nowadays, proteomics coupled with bioinformatics analysis is increasingly used in biological research to fully understand the potential targets and signaling pathways [8]. The proteomic platform provides a powerful tool for us to perform high-throughput studies allowing the detection of modulated proteins in response to natural compound treatment. In particular, the shotgun approach based on one-dimensional (1D) PAGE with liquid chromatography chip and Q-TOF MS/MS allows the qualitative and quantitative analysis of a large number of proteins in complex samples, avoiding two-dimensional electrophoresis (2-DE) and isotopic labeling [9]. The information offered by proteomics provides context-based understanding of protein networks and such an investigative approach is rendered more insightful by the support of bioinformatics tools, which may highlight the main cellular pathways altered in the samples under investigation [10].

In the present study, we investigated the effects of 6-shogaol on cell viability and apoptosis mechanisms in human cervix adenocarcinoma HeLa cells for the first time. A shotgun proteomic approach was applied to profile the differential protein expression in the HeLa cells after 6-shogaol treatment. To further validate the proteomic data, we performed western blot analysis on several selected differentially expressed protein. A comprehensive signaling network analysis was conducted to uncover the protein-protein functional association. The protein profilings were characterized with annotated protein cellular location and biological process to further understand the molecular mechanism of HeLa cells treated with 6-shogaol. 

## 2. Materials and Methods

### 2.1. Materials and Regents

6-Shogaol was isolated from *Z. officinale* and purified by a series of chromatography procedures in our laboratory as reported earlier [11]. Their structures were elucidated by comparison of spectral data (UV, MS, and NMR) with the literature data. Its purity was determined as higher than 95% by TOF/MS. 6-Shogaol was dissolved in DMSO and stored at −80°C.

Acetonitrile (ACN) was purchased from Merk (Darmstadt, Germany). Nuclease mix was from Amersham Bioscience (Uppsala, Sweden). Other chemical regents, except for specially noted, were obtained from Sigma (St. Louis, USA). Antibodies for Annexin A1 and cofilin were purchased from Abcam (Cambridge, MA). Antibodies for calreticulin, PERK, CHOP, ARF5, HSP60, PARP, Pro caspase 3, Bax, *β*-Actin and Horseradish peroxidase (HRP)-conjugated secondary goat anti-mouse or anti-rabbit antibodies were from Cell Signaling Technology (Beverly, MA). Anti-Tubulin was from Anbo Biotechnology Company (San Francisco, CA, USA).

### 2.2. Cell Culture

The human cervical cancer cell line HeLa was purchased from American Type Culture Collection (Manassas, VA), maintained in Dulbecco's modified Eagle's medium-high glucose supplemented with 10% (v/v) heat-inactivated fetal bovine serum (GIBCO-BRL, Grand Isle, NY), 100 U/mL penicillin and 100 *μ*g/mL streptomycin. Cells were kept in a humidified 5% CO_2_ incubator at 37°C. When the cells grew to about 70-80% confluence, they were subcultured.

### 2.3. Cell Proliferation Assay

The antiproliferative effect of 6-shogaol against HeLa was determined by the 3-(4,5- dimethylthiazol-2-yl)-2,5-diphenyltetrazolium bromide (MTT) assay [12]. Briefly, cells (5 × 10^3^ per well) were seeded in 96-well plates overnight for attachment and treated with 6-shogaol in different concentrations for 24 h. Doxorubicin (8.6 *μ*M) was used as positive control. Then twenty microliters of MTT solution (5 mg/mL in PBS) was added to each well, and the cells were further incubated for 4 h. The medium was aspirated, and the purple formazan crystals were dissolved by adding 200 *μ*L DMSO. After shaking gently, absorbance at 570 nm was measured by microplate enzyme-linked immunosorbent assay reader. The IC_50_ was calculated by nonlinear regression analysis. Each experiment was repeated a minimum of three times using three wells per drug concentration. 

### 2.4. Morphologic Changes

For detection of an apoptotic body, cells were judged according to their nuclear morphology and the disintegration of their cell membranes. HeLa cells were plated at a density of 5 × 10^5^/well in 6-well plate, 12 h later, 15 *μ*M of 6-shogaol was added to the media and cells were incubated for 12 h. At the end of the culture period, cells were washed with PBS, and were stained with Hoechst 33342 (1 *μ*g/mL) for 15 min at 37°C. After a final wash in PBS, samples were visualized under Nikon fluorescence microscopy [13]. 

### 2.5. Flow Cytometric Analysis of Apoptosis and Cell Cycle

Apoptosis and cell cycle distribution in HeLa cells were measured at 24 h after treatment with 15 *μ*M of 6-shogaol. The cells for apoptosis analysis were harvested, washed twice with ice-cold PBS, and then resuspended with Annexin V-FITC Apoptosis Detection Kit (Beyotime, China) according to manufacturer's instructions. The cells for cell-cycle analysis were fixed with 70% ethanol at −20°C and then washed in ice-cold PBS and resuspended in RNaseA (10 mg/mL) and propidium iodide (PI, 50 mg/mL) overnight as reported [14]. Analyses were applied on a FACS auto flow cytometer (BD Biosciences; Mountain View, CA). Annexin V+/PI− cells were considered as early apoptotic while Annexin V+/PI+ cells as late apoptotic or necrotic. The percentages of apoptotic cells for each sample were estimated with FACScan software (BD Biosciences; Mountain View, CA), while the number of cells in G0, G1/S, and G2/M phases was calculated using Modfit LT software (BD Biosciences; Mountain View, CA).

### 2.6. Western Blot Analysis

Cells in serum-free medium were incubated with or without 6-shogaol (15 *μ*M) for designated times. After scraped from the 10 cm dishes the cells were washed twice with PBS and lysed with cell lysis buffer (Beyotime, China) containing 1 mM phenylmethylsulfonyl fluoride (PMSF) on ice for 30 min. Then the whole cell extracts were centrifuged at 13,000 ×g for 10 min. The supernatant was collected and stored at −20°C. The protein content of the cell extracts was determined by the Bradford assay (Bio-Rad, USA). Proteins were separated on 12% SDS- polyacrylamide gel. After electrophoresis, the proteins were transferred to a nitrocellulose membrane, blocked with 5% nonfat powdered milk for 2 h, and the membranes were incubated with primary antibodies overnight at 4°C. The blots were washed with TBST three times and then probed with HRP-conjugated secondary antibodies for 2 h at room temperature. Proteins were visualized by the enhanced chemiluminescence detection system (ECL, Millipore) and exposed to Kodak autoradiography film as reported [15]. *β*-Actin was used as the internal control.

### 2.7. Caspase 3 Activity Assay

The activity of caspase-3 assay was determined according to Caspase-3 Activity Assay Kit (Beyotime, China). The caspase-3 activity is measured based on spectrophotometric detection of the cleavage of the acetyl-Asp-Glu-Val-Asp p-nitroanilide (Ac-DEVD-pNA) substrate to chromophore p-nitroanilide (pNA) [16]. According to the manufacturer's protocol, HeLa cells were lysed after treatment with 6-shogaol (15 *μ*M). Assays were carried out on 96-well plates by incubating 30 *μ*L of cell lysate, 60 *μ*L of reaction buffer, and 10 *μ*L of caspase-3 substrate (Ac-DEVD-pNA) (2 mM) per sample. Lysates were incubated at 37°C overnight. Samples were measured with an ELISA reader at an absorbance of 405 nm. Relative caspase 3 activity was calculated as a ratio of emission of 6-shogaol treated cells to the control group. All the experiments were performed in triplicates.

### 2.8. Measurement of Mitochondrial Transmenbrane Potential

Mitochondrial transmembrane potential (ΔΨm) was measured by JC-1 mitochondrial membrane potential assay kit (Beyotime, China) and performed following manufacturer's protocol. After indicated treatment, the cells cultured in six-well plate were incubated with JC-1 (5 *μ*g/mL) at 37°C for 20 min in the dark. Then we monitored the mitochondrial membrane potentials by determining the relative amounts of dual emissions from mitochondrial JC-1 monomers (green) or aggregates (red) using a Nikon eclipseTi-S fluorescent microscope. Mitochondrial depolarization was shown by an increased intensity of green/red fluorescence.

### 2.9. Protein Extraction, Separation, and Digestion

As reported before [17], cells were treated with 0.1% DMSO or 15 *μ*M 6-shogaol for three independent experiments. After treatment for 24 h, the pooled cells were collected. 5 × 10^6^ cells were suspended in 100 *μ*L of lysis solution and put in an ice-cold supersonic bath for 30 min. After centrifugation, the supernatant was collected and the protein concentration was determined using the Bradford protein assay kit (Bio-Rad, USA). After being separated by SDS-PAGE, the PAGE was stained with Coomassie brilliant blue G-250 and cut into slices. Before MS analysis, the gel was destained with 100 mM ammonium bicarbonate (ABC)/50% ACN (1 : 1, v/v), and ACN was added to dehydrate. Subsequently, trypsin (10 ng/*μ*L) was added to the gel pieces, and then ABC/ACN (9 : 1, v/v) of 40 mM was added to cover, and digested overnight. Then the digest pieces were extracted with a solution containing 50% ACN and 5% formic acid (FA). The digested peptides were concentrated by freeze drier and stored at −80°C for further MS analysis.

### 2.10. LC-CHIP Q-TOF MS/MS Analysis

The LC-CHIP Q-TOF MS/MS analysis was conducted as described in our previous report [17]. Briefly, the lyophilized peptides were resuspended in 0.1% FA. The resuspended peptide solution was enriched and fractionated with HPLC-Chip (Agilent 1200 Series HPLC systems). The software HPLC-Chip Cube MS interface was used to control the whole analytical process. A total of 1.0 *μ*L sample (200 ng) was injected into enrich column to desalt and analyze online through MS^n^ after isocratic eluted and gradient eluted by enrich and separate columns, respectively. The peptides were loaded into the enrichment column before analytical separation. Agilent 6520 ESI Q-TOF Mass Spectrometer adopted Chip cube was used as ion source. Precursor selection selected 3 max precursors per cycle, active exclusion by enabled mode, excluded after 1 spectrum and released after 0.25 min and the MS or MS/MS scanning range of *m/z *from 300 to 3000 or 100 to 3000. 

### 2.11. Database Search for Protein Identification

Database search was applied as previous reported [17]. Briefly, the data of MS and MS/MS were analyzed using Agilent G2721AA Spectrum Mill MS Proteomics Workbench (Rev A.03.03.078) against the database of UniProtKB/SWISSProt, species of Homosapiens (human). The value of peptide spectral intensity was obtained from the analyzed data of MS and MS/MS by spectrum mill proteomics workbench. The Spectrum Mill Data Extractor program prepared MS/MS data files for processing. Autovalidation was carried out after searching by calculated reversed database scores to rule out false positives.

### 2.12. Bioinformatics Analysis

To classify the identified proteins, bioinformatics tools were used to obtain information on protein annotation, protein-protein interactions, and their potential pathway. An analysis of molecular function and biological process was performed using PANTHER classification system (version 7.2, http://www.pantherdb.org/), which is a unique resource using evolutionary relationships and published scientific experimental evidence to predict functions [18]. To identify the signaling pathways towards changed proteins, a search tool for the retrieval of interacting genes/proteins (STRING, version 9.0) was used to connect the associated proteins. Only interactions with a confidence score more than 0.7 and exclusively based on experimental or database information were considered for protein network visualization [19, 20]. In addition, ingenuity pathway analysis (IPA; Ingenuity Systems, http://www.ingenuity.com/) tool was applied to identify interaction networks and predominant canonical pathways. The scores associated with each form of analysis were calculated using logarithm of the *P* value (Fisher's exact test), indicating the likelihood that the altered proteins would be found in a given network by chance. *P* < 0.05 was set as significance, which represents the probability that these proteins are connected in one network just due to chance alone are significantly small [21]. 

### 2.13. Statistical Analysis

Presented data are the mean ± SD values for the indicated number of independent experiments. Statistical differences between groups were calculated using Student's two-tailed *t*-test (GraphPad Prism). Differences were considered statistically significant for values *P < 0.05, **P < 0.01.

## 3. Results

### 3.1. Cytotoxic Effects by 6-Shogaol Treatment in Human Cervical Cancer Cells

**Figure 2:**
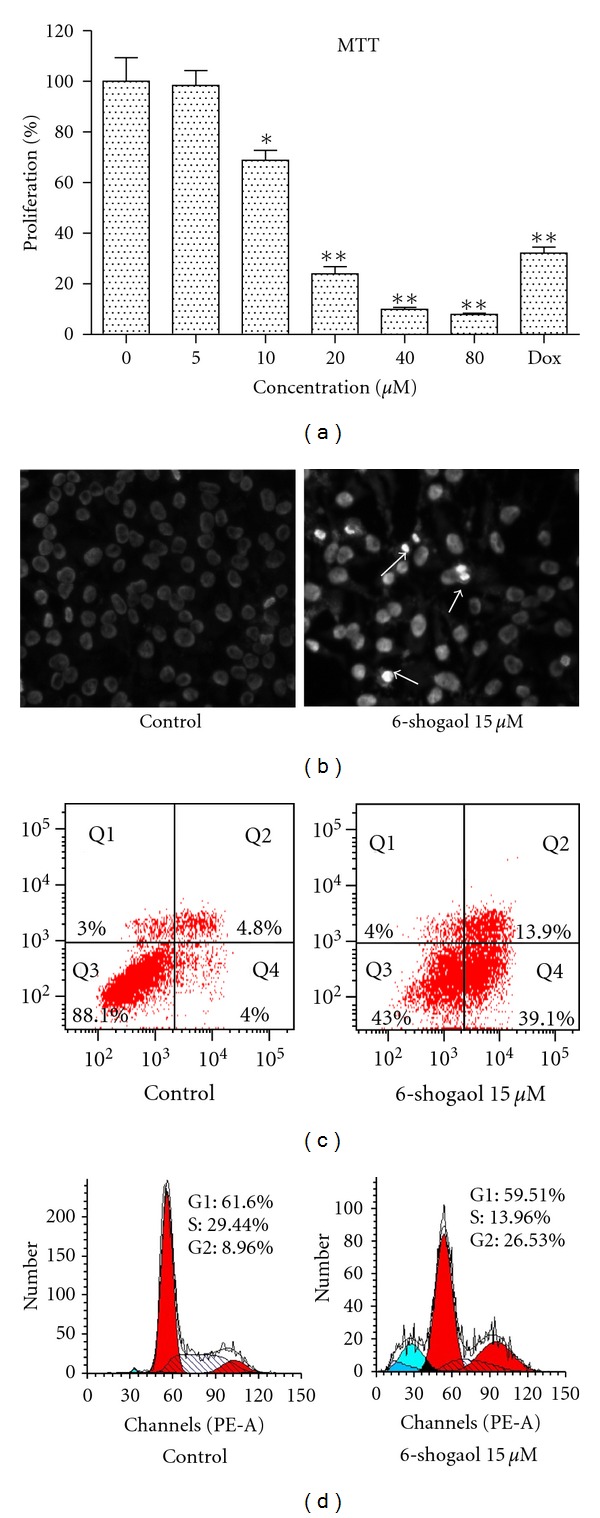
Effects of 6-shogaol on cervical cancer cell proliferation, apoptosis, and cell-cycle arrest. (a) HeLa cells were treated with 0.1% DMSO, 5, 10, 20, 40, and 80 *μ*M 6-shogaol and 8.6 *μ*M doxorubicin for 24 h, and cell viability was determined by MTT assay. (b) Morphological change induced by 15 *μ*M 6-shogaol in HeLa cells after 24 h treatment (1200x magnification). Typical apoptotic morphological change in 6-shogaol treated cells was observed. Apoptosis (c) and DNA histograms (d) of HeLa cells were obtained by flow cytometry analysis. Apoptosis and accumulation in G2/M phase was observed after 24 h of 6-shogaol treatment. An increase in the percentage of apoptotic cells was also obtained. Shown are representative results of three independent experiments.

To evaluate the effect of 6-shogaol on cell growth, proliferation assays were performed using HeLa cells, with increasing drug concentrations (5, 10, 20, 40, 80 *μ*M) and cell viability was determined by the MTT assay. As shown in Figure 2(a), 6-shogaol significantly inhibited HeLa proliferation in a dose-dependent manner. The IC_50_ was 14.75 ± 0.94 *μ*M when HeLa cells were treated with 6-shogaol for 24 h. To determine whether the anti-proliferative activity induced on HeLa cell line by 6-shogaol was mediated by induction of apoptosis and cell-cycle arrest, we evaluated apoptosis by morphological examination under fluorescence microscopy and conducted cell-cycle distribution analysis by flow cytometry. Typical features of apoptosis like chromatin condensation and nuclear fragmentation of 6-shogaol-treated cells were clearly observed from fluorescence microscopic studies (Figure 2(b)). The apoptosis rate (early and late apoptosis) was 53.0% in 6-shogaol-treated group, compared with only 8.8% in the control group (Figure 2(c)). A significant increase in G2 peak was observed in 6-shogaol-treated HeLa cells (Figure 2(d)), suggesting that 6-shogaol could induce cell-cycle arrest in G2 phase.

### 3.2. 6-Shogaol-Induced Endoplasmic Reticulum Stress and Apoptosis through Mitochondrial Pathway and Induced Loss of Mitochondrial Membrane Potential of HeLa Cells

**Figure 3:**
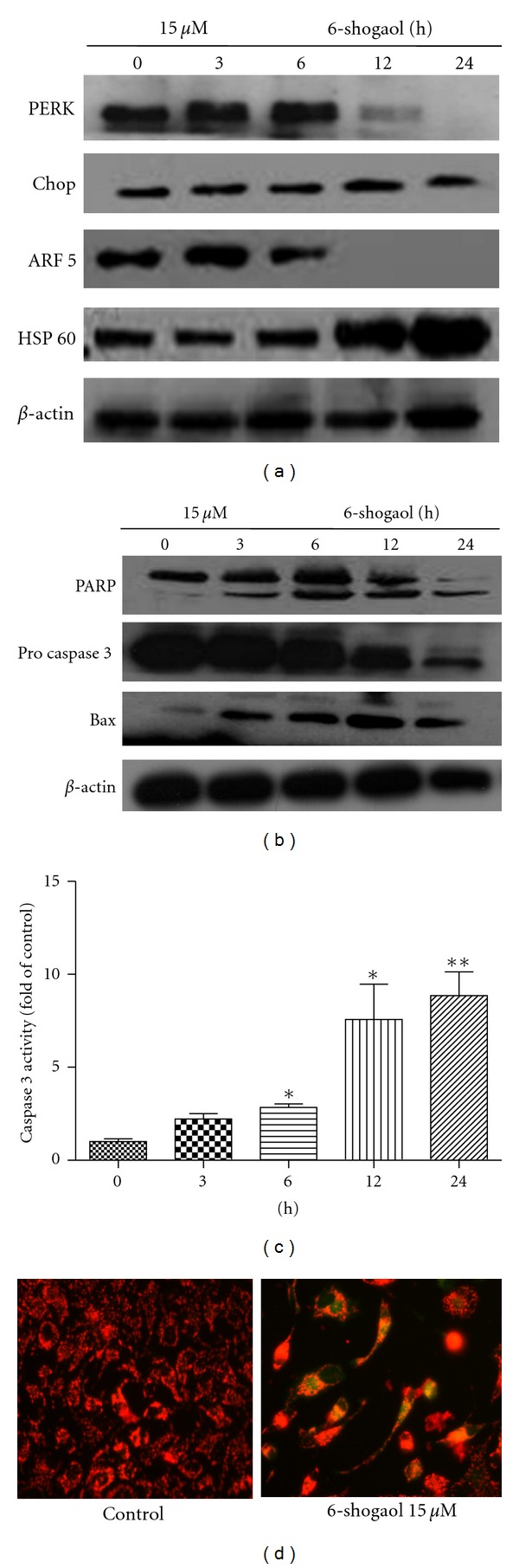
Effects of 6-shogaol on expression of proteins on ER stress and mitochondrial apoptosis pathway, as well as the dissipation of ΔΨm on HeLa cells. The levels of PERK, CHOP, ARF5, HSP60 (a), PARP, Pro Caspases-3, and Bax (b). Normalization performed to *β*-actin. The bands shown here were from a representative experiment repeated three times. (c) The caspase 3 activity was detected according to Caspase 3 Activity Assay Kit. Relative caspase-3 activity was calculated as a ratio of emission of 6-shogaol treated cells to vehicle-treated (0.1% DMSO) cells. Values were means ± SD of three independent experiments. *(*P* < 0.05); **(*P* < 0.01), compared with control cells. (d) Red fluorescence represents the mitochondrial aggregate form of JC-1, indicating dissipation of ΔΨm. Cells subjected to 15 *μ*M of 6-shogaol were stained by JC-1. Change of ΔΨm was detected by fluorescence microscopy. Vehicle-treated cells which have high ΔΨm show punctuate red fluorescence. Apoptosis cells show diffuse green fluorescence because of decrease in mitochondrial membrane potential.

To examine the effect of 6-shogaol on the apoptotic signaling pathway, the levels of the ER-stress-associated proteins PERK, CHOP, ARF5, and HSP60, and mitochondrial apoptosis-associated proteins PARP, Bax, and pro caspase-3 were detected by Western blot. As shown in Figure 3(a), 6-shogaol decreased the protein expression of PERK and ARF5 but stimulated HSP60 expression. In contrast, exposure to 6-shogaol resulted in little or no change in expression of CHOP. The results suggest that ER stress may play a critical role in 6-shogaol lethality in HeLa cells.

Bcl-2 family proteins, such as Bax, are critical in regulating apoptosis [22]. 6-Shogaol increased the protein expression of Bax in a time-dependent manner as shown in Figure 3(b). Caspase family proteins play an important role in executing apoptosis. Caspase-3, the main executioner for apoptosis, can be activated by upstream initiator caspase-9 through intrinsic apoptosis pathway [23]. Our study showed that treatment with 6-shogaol resulted in activation of caspase 3 in a time-dependent manner. (Figures 3(b) and 3(c)). DNA repair enzyme PARP is the downstream substrate of activated caspase-3/7 and cleavage of PARP is associated with a variety of apoptotic responses [23]. Also, cells treated with 6-shogaol displayed cleaved signature peptide of PARP. 

Previous reports have demonstrated that early apoptosis is always accompanied by the irreversible loss of mitochondrial membrane potential [24]. In this study, we investigated the effect of 6-shogaol on mitochondrial membrane potential using JC-1. As shown in Figure 3(d), an increased intensity of green/red fluorescence was observed, which indicated that 15 *μ*M of 6-shogaol could significantly cause the collapse of mitochondrial membrane potential. These findings suggest that 6-shogaol destroys the mitochondrion function of HeLa cells to induce apoptosis.

### 3.3. Shotgun Proteomic Analysis of the Protein Profiling

In order to get insight into the protein expression profile of HeLa cells in response to 6-shogaol exposure and to further explore the molecular mechanisms, we employed a label-free shotgun proteomic technique to profile proteome changes between vehicle control and drug treatment. In our previous study, the reproducibility of this method had been evaluated, and a reliability coefficient of 95% was shown among independent experiments. Also we found that the parameter of peptide spectral intensity was better than spectra counts in shotgun proteomics protocol's quantification [17]. Consequently, the average peptide spectral intensity was determined as the standard to relatively quantify the target-specific proteins. Utilizing Spectrum Mill MS Proteomics Workbench, a total of 287 proteins whose expressions were significantly changed under 6-shogaol treatment were identified, in which 76 proteins (54 upregulated and 22 downregulated) showed more than 2-fold changes peak intensity compared with the control. The list of identified proteins, gene names, accession number, MW/pI, coverage, scores unique, cellular component, biological process and fold change were shown in Table  S1 in Supplementary Material available online at doi:10.1155/2012/278652 (upregulation) and Table S2 (downregulation). The identified proteins were distributed at different locations in the cell component as the table showed.

**Figure 4:**
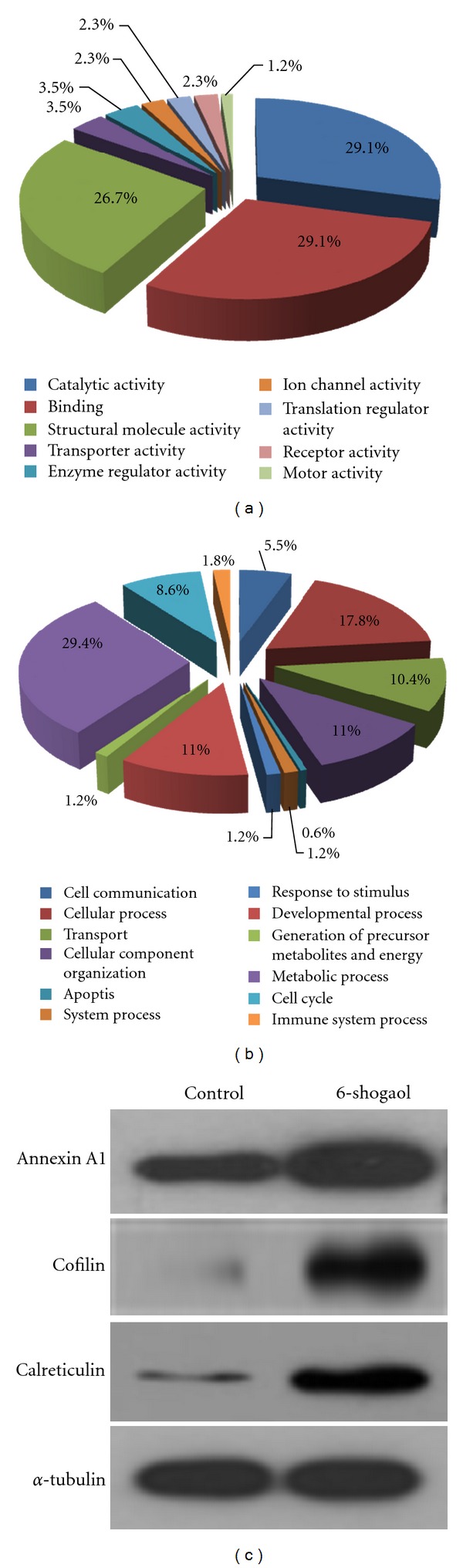
GO categorization of the 6-shogaol-regulated proteins and the validation of differentially expressed proteins. Based on the PANTHER classification and gene ontology (GO) categorization, molecular function (a) and biological process (b) of the corresponding identified proteins were classified. (c) The differentially expressed proteins Annexin A1, cofilin, and calreticulin were validated by Western blot. Control and 6-shogaol marked above the panel represent the control cells and cells treated with 15 *μ*M of 6-shogaol for 24 h. Tubulin was used to normalize protein loading. Each blot is the representative result of three independent experiments.

In order to better understand the differentially expressed proteins, a GO analysis with PANTHER classification system was used to analyze their molecular functions and biological process. Based on PANTHER protein class analysis, the modulated proteins were classified into 9 groups according to their molecular function, that is, catalytic activity (29.1%), binding (29.1%), and structural molecule activity (26.7%). By their biological process, the modulated proteins could be divided into 12 groups including apoptosis, cell cycle, cellular component organization, and so forth (Figures 4(a) and 4(b)). The 6-shogaol-regulated proteins firstly found by proteomic assay may provide us crucial clues for future research on 6-shogaol-induced cytotoxic effect.

To validate the proteome data, we randomly selected three proteins with higher fold change values, including Annexin A1, cofilin and calreticulin, and assessed their expression between vehicle control and 6-shogaol treatment by Western blot. Comparing the treatment group to the control, Figure 4(c) demonstrates clearly that these three differentially expressed proteins were remarkably upregulated. Hence, the results of western blot analysis confirmed the reliability of the proteomic analysis.

### 3.4. Construction of the Signal Network and Prediction of the Pathway of 6-Shogaol

**Figure 5:**
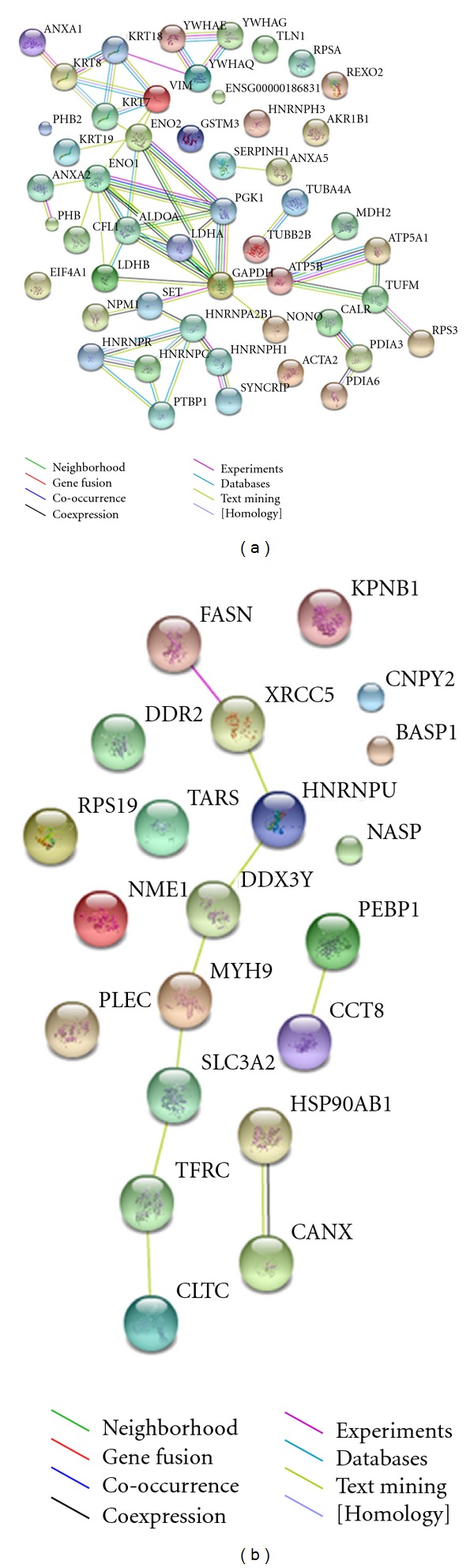
Construction of integrated signaling networks. The proteins that are upregulated (a) and downregulated (b) from proteomic analysis were uploaded to the STRING tool to identify functional signaling networks. The protein-protein interaction network is presented by GO classification. The predicted functional links consist of up to eight lines: one color for each type of evidence.

**Figure 6:**
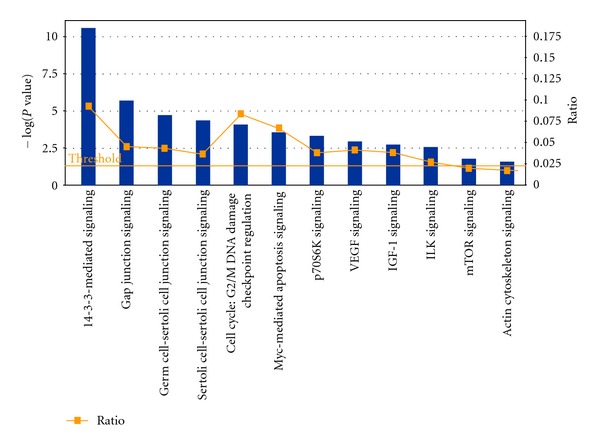
The canonical pathways focus on the signaling pathways that were modulated in 15 *μ*M 6-shogaol-treated HeLa cells as analyzed by IPA software (Ingenuity Systems, http://www.ingenuity.com/). The *x*-axis represents the pathways identified. The *y*-axis (left) shows the −log of the *P* value calculated based on Fisher's exact test. The ratio (*y*-axis, right) represented by the orange points is calculated as follows: numbers of genes in a given pathway that meet cutoff criteria, divided by total numbers of genes that make up that pathway. The orange line stands for the threshold above which there are statistically significantly (by default *P* < 0.05).

**Figure 7:**
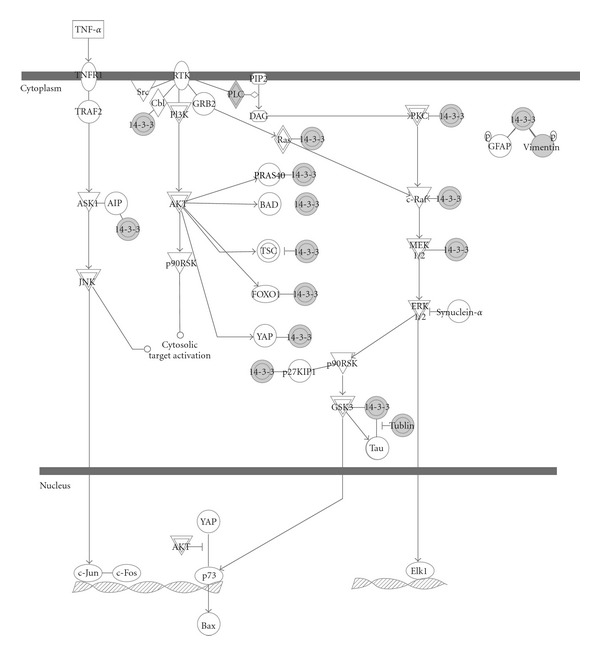
14-3-3 signaling canonical pathway from IPA. The analysis data showed that the response proteins 14-3-3 (SFN, YWHAE, YWHAG, and YWHAQ), Tubulin (TUBA4A, TUBB3, TUBB, TUBB2B, and TUBB4B), PLC (PDIA3), and VIM were involved in this pathway. Complex are represented by a shape with a double-line.

Moreover, to understand the cellular pathways involved in 6-shogaol-treated cervical cancer cells, we performed an unsupervised bioinformatics analysis using the web-based tool STRING. The network was produced by the shortest path algorithm to map the interacted proteins. As the high-level functional linkage of proteins may facilitate the prediction of the whole biological processes, we set the high confidence required score to more than 0.700. To analyze the connection among these huge numbers proteins clearly, we first separated them into two parts. As illustrated in Figure 5, the upregulated (Figure 5(a)) and downregulated proteins (Figure 5(b)) were integrated into interconnected protein networks. In the network, some proteins with consistent changes interact with each other, that is, VIM-KRT18-YWHAQ-YWHAE and PHB-ANXA2-ENO1-LDHA in upregulated proteins, CANX-HSP90AB1 and HNRNPU-DDX3Y-MYH9 in downregulated proteins. Both networks were generated by most of the differently expressed proteins identified, which were connected directly or indirectly. In order to further identify the potential pathway of 6-shogaol and explore its biological mechanism, the Swiss-Prot accession numbers of the 76 modulated proteins were completely uploaded for analysis using IPA software. A Fisher's exact test was applied to determine the relationship between a specific pathway and the proteins uploaded. Consequently, four major networks were generated with the major function; (1) cancer, cellular assembly and organization, and cellular function and maintenance, (2) DNA replication, recombination, and repair, cancer and hematological disease, (3) molecular transport, nucleic acid metabolism and small molecule biochemistry, (4) cellular movement, skeletal and muscular system development and function and cell-to-cell signaling.  Figure 6 shows the signaling canonical pathways associated with the above networks (*P* < 0.05, fisher's exact test). The top canonical pathway matched in IPA was “14-3-3 mediated signaling” (Figure 7), which involved 11 nodes, including 14-3-3 (SFN, YWHAE, YWHAG and YWHAQ), Tubulin (TUBA4A, TUBB3, TUBB, TUBB2B and TUBB4B), PLC (PDIA3), and VIM. The canonical pathways analysis highlighted one series of molecular, 14-3-3, which may play a central role in exerting the multiple biological mechanism of 6-shogaol.

## 4. Discussion

Over the years, there has been a worldwide interest in ginger as a dietary supplement because of its various health beneficial effects. Shogaols, the dehydrated form of gingerols exist in higher amounts in dried ginger [25], have been reported to possess antitumor effects and to date, findings have revealed that they are likely to induce cancer cell death via multiple mechanisms. Earlier studies suggested that anticancer effects of 6-shogaol were associated with apoptosis, autophagy, antiinvasion, and antiproliferation [6, 7, 26]. However, so far as we know, there is no available information concerning 6-shogaol's *in vivo* efficacy against cervical cancer. In the present study, we have demonstrated that 6-shogaol could significantly inhibit the growth of HeLa human cervical cancer cells. Consistent with findings of studies on other cancer cell lines [7], the results of the morphology and flow cytometry assay indicated that 6-shogaol arrested cell-cycle at G2/M phase and triggered apoptosis. We have also observed that 6-shogaol caused the activation of Bax, caspase-3, and the degradation of PARP. It is well known that the cascade events of the apoptotic process always led to the depletion in mitochondrial transmembrane potential, and consequently resulted in the activation of caspase 3. Consistent with the Pan's report in COLO 205 cells [27], the change of ΔΨm in 6-shogaol-treated cells in the present study, demonstrated that the mitochondrial apoptosis pathway might be involved in 6-shogaol lethality.

Proteomics is a useful technique for identifying targeted metabolic or signaling pathways especially when changes in protein expression can be related to physiological and biochemical parameters that respond to the treatment of interest. As the molecular mechanism in which 6-shogaol induces proliferation inhibition, apoptosis, and cell-cycle arrest is still not clear, we implemented the proteomics scheme to search globally for the differentially expressed proteins in HeLa cells affected by 6-shogaol. Label-free shotgun quantitative strategies are attractive alternatives for quantitative LC/MS/MS-based proteomics because of their simplicity, affordability, and flexibility. Herein, we analyzed the label-free shotgun proteomic alterations occurring in the HeLa cells after exposure to 6-shogaol LC Chip Q-TOF/MS. Totally, 65 signal related proteins with more than two-fold changes were identified. Function analysis suggests that the alteration proteins after 6-shogaol treatment are associated with catalytic activity, binding, and structural molecule activity. Consistent with the results of previous cytotoxic assays, some of the 6-shogaol-regulated proteins are apoptosis and cell-cycle associated proteins classified by PANTHER. Three up-regulated binding proteins including Annexin A1, calreticulin and cofilin, were validated by Western blot analysis in the present study. Annexin A1 has multifunctions, including the inhibition of the NF-*κ*B signal transduction pathway and the enhancement of apoptosis, as well as regulating cell growth [28, 29]. It was reported that decreased expression of Annexin A1 was found during the progression of cervical neoplasia [30]. In the present study, we observed that 6-shogaol dramatically increased the expression of Annexin A1, which may help to explain the antiproliferation effect of 6-shogaol. Calreticulin, a multifunctional protein binds to Ca^2+^ ions, is located in storage compartments associated with endoplasmic reticulum. It is also known that incapable of activating the Calreticulin exposure pathway is refractory to the anticancer therapies [31]. The upregulation of Calreticulin after 6-shogaol treatment in HeLa cells confirmed by immunoblotting analysis was in accordance with findings reporting the ability of curcumin to induce the unfolding protein response by upregulating the Calreticulin expression [32].

As data visualized in networks can be used to clearly interpret the proteins interactions, we combined the proteomic assay and bioinformatics analysis, which are popularly used in integrating and mining proteomic data, to explore the cytotoxic mechanism of 6-shogaol. Here, we applied the commercial Ingenuity Pathway Analysis tool as well as the online software STRING to map the whole protein profile. Based on the STRING analysis, a well-connected network which showed a reliable relationship among the response proteins associated directly or indirectly was constructed. Moreover, IPA tool was applied to further investigate the underlying pathway. Four key networks were identified to be involved in a variety of signaling and metabolic pathways. The top canonical signaling pathway was the “14-3-3 mediated signaling.” The 14-3-3 protein family, including seven 14-3-3 isoforms (beta/alpha, epsilon, gamma, eta, theta/tau, sigma, and zeta/delta), is a family of phosphoserine/phosphothreonine binding proteins. It was reported that 14-3-3 proteins play an important role in the regulation of wide variety biological process, including proliferative, cell cycle and apoptotic signaling [33]. As shown in Figure 7, binding of 14-3-3 to Raf, a key effector to initiate the activation of the extracellular signal-regulated kinase (ERK) cascade will later influence proliferative signaling. In our current proteomic study, 14-3-3 proteins including epsilon, gamma, sigma, and theta were significantly upregulated. 14-3-3 proteins were reported to bind the critical proapoptotic effector Bax, which is overexpression after 6-shogaol treated [34]. Additionally, 14-3-3 sigma, acts as a tumor suppressor, was found to be downregulated in breast cancers. Otherwise, 14-3-3 sigma could bind the cdc2-cyclin B1 complex in the cytoplasm resulting in G2 arrest [35]. Also two cell-cycle-associated proteins BRCA1 and p53, which exert their function partly on the maintenance phase at G2/M, will lead to the expression of 14-3-3 sigma [36]. Yue et al. found Ganoderic Acid D, a compound isolated from *Ganoderma lucidum*, induced cytotoxic effect on HeLa mainly through 14-3-3 family [37], which is consistent with our results.

Apart from the signaling pathways, a series of metabolic pathways are found to be involved in the networks, and the most highlighted one is “glycolysis/gluconegensis” (data not shown). Also, IPA diseases analysis data showed 8 proteins were associated with cervical cancer, including ANX1, ANX2, ANX 5, ENO1, HSP90AB1, YWHAE, TFRC, HSP90, and KRT19. Whether these proteins play a crucial role in the cytotoxic mechanism of 6-shogaol needs further investigation.

It must be noted that some proteins associated with apoptosis detected in western blot assay were not identified in proteomic analysis. We believed that it may be caused by the different lysis buffers used in immunoblotting and proteomic assay, which influenced the solubility of some proteins and further led to identification differentiation. Additionally, electrophoresis and label-free comparative proteomic is different in methodology level. So the changed proteins identified in both experiments cannot overlap each other completely.

In summary, this is the first report, to our knowledge, describing the mechanism of 6-shogaol-mediated inhibition of cervical cancer cell growth using a proteomic approach. It is conceivable that 6-shogaol exerts multiple stimuli to several biological pathways in HeLa cells. Interestingly, the results of comprehensive signaling network analysis suggested that 14-3-3 signaling might play the important role in the cytotoxic mechanism of 6-shogaol. The differentially expressed proteins identified by label-free shotgun proteomic technique will be further investigated as potential targets of 6-shogaol. Taken together, these results shed light on the anticancer mechanism of 6-shogaol from a molecular perspective.

## Supplementary Material

76 proteins including 54 up-regulated and 22 down-regulated ones with more than 2-fold changed have been listed in supplementary materials. The contend of lists including protein names, gene names, accession number, MW/pI, coverage, scores unique, cellular component, biological process and fold changes. The protein information was obtained from the database of UniProtKB/SWISSProt.
