# Supplementary material for: The Cytotoxicity Mechanism of 6-Shogaol-Treated HeLa Human Cervical Cancer Cells Revealed by Label-Free Shotgun Proteomics and Bioinformatics Analysis
Source: Evid Based Complement Alternat Med. 2012 Nov 11;2012:278652. doi: 10.1155/2012/278652 (PMC3518257; doi:10.1155/2012/278652)
Supplement: Supplementary file 1 — 76 proteins including 54 up-regulated and 22 down-regulated ones with more than 2-fold changed have been listed in supplementary materials. The contend of lists including protein names, gene names, accession number, MW/pI, coverage, scores unique, cellular component, biological process and fold changes. The protein information was obtained from the database of UniProtKB/SWISSProt. [file 278652.f1.doc]

**Supplementary data**

**Table S1** Identification of up-regulated proteins in 6-shogaol treated HeLa cells using LC Chip Q-TOF MS/MS.

| No. | protein name | gene name | Swiss  Prot | Pro.  MW | Pro.  pI | Cov.* | S.U.^†^ | cellular component | biological process | FC^‡^ |
| --- | --- | --- | --- | --- | --- | --- | --- | --- | --- | --- |
| 1 | Protein disulfide-isomerase A3 | PDIA3 | P30101 | 56782.7 | 5.99 | 20 | 126.5 | ER | protein retention in ER | 43.97 |
| 2 | Annexin A5 | ANXA5 | P08758 | 35936.9 | 4.94 | 37 | 204.43 | [cytoplasm](http://www.ebi.ac.uk/ego/DisplayGoTerm?id=GO:0005737) | [anti-apoptosis](http://www.ebi.ac.uk/ego/DisplayGoTerm?id=GO:0006916),[blood coagulation](http://www.ebi.ac.uk/ego/DisplayGoTerm?id=GO:0007596) | 42.27 |
| 3 | Aldose reductase | AKR1B1 | P15121 | 35853.6 | 6.52 | 28 | 110.32 | [cytoplasm](http://www.uniprot.org/keywords/KW-0963), [nucleus](http://www.ebi.ac.uk/ego/DisplayGoTerm?id=GO:0005634) | [response to stress](http://www.ebi.ac.uk/ego/DisplayGoTerm?id=GO:0006950) alditol | 21.83 |
| 4 | Nucleophosmin | NPM1 | P06748 | 32575.2 | 4.64 | 19 | 59.86 | [nucleus](http://www.uniprot.org/keywords/KW-0539) | [DNA repair](http://www.ebi.ac.uk/ego/DisplayGoTerm?id=GO:0006281), [anti-apoptosis](http://www.ebi.ac.uk/ego/DisplayGoTerm?id=GO:0006916) | 16.25 |
| 5 | Annexin A1 | ANXA1 | P04083 | 38714.5 | 6.57 | 41 | 223.29 | [cell membrane](http://www.uniprot.org/keywords/KW-1003) | [anti-apoptosis](http://www.ebi.ac.uk/ego/DisplayGoTerm?id=GO:0006916) | 15.65 |
| 6 | Heterogeneous nuclear ribonucleoproteins C1/C2 | HNRNPC | P07910 | 33670.2 | 4.95 | 14 | 62.98 | [nucleus](http://www.uniprot.org/keywords/KW-0539) | [mRNA processing](http://www.uniprot.org/keywords/KW-0507), [mRNA splicing](http://www.uniprot.org/keywords/KW-0508) | 15.22 |
| 7 | Prohibitin-2 | PHB2 | Q99623 | 33296.5 | 9.83 | 23 | 85.9 | [mitochondrial inner membrane](http://www.ebi.ac.uk/ego/DisplayGoTerm?id=GO:0005743) | [Transcription regulation](http://www.uniprot.org/keywords/KW-0805) | 14.48 |
| 8 | Malate dehydrogenase MDH2,mitochondrial | MDH2 | P40926 | 35531.5 | 8.92 | 22 | 103.03 | [mitochondrion](http://www.uniprot.org/keywords/KW-0496) | [Tricarboxylic acid cycle](http://www.uniprot.org/keywords/KW-0816) | 13.20 |
| 9 | Heterogeneous nuclear ribonucleoproteins A2/B1 | HNRNPA2B1 | P22626 | 37429.9 | 8.97 | 16 | 89.13 | nucleus, splicesome | RNA transport, RNA splicing | 12.02 |
| 10 | 14-3-3 protein epsilon | YWHAE | P62258 | 29174.1 | 4.63 | 23 | 93.25 | melanosome, cytoplasm | intracellular signaling cascade | 10.81 |
| 11 | Serpin H1 | SERPINH1 | P50454 | 46440.8 | 8.75 | 21 | 101.53 | ER | response to stress, protein folding | 8.06 |
| 12 | Annexin A2 | ANXA2 | P07355 | 38604.2 | 7.58 | 49 | 359.89 | plasma membrane | skeletal development | 7.81 |
| 13 | 40S ribosomal protein S3 | RPS3 | P23396 | 26688.5 | 9.68 | 21 | 80.99 | Cytoplasm | ribosome translation | 7.49 |
| 14 | Fructose-bisphosphate aldolase A | ALDOA | P04075 | 39420.2 | 8.30 | 27 | 134.32 | actin cytoskeleton, cytosol | glycolysis fructose metabolic process | 7.35 |
| 15 | Elongation factor Tu, mitochondrial | TUFM | P49411 | 49541.8 | 7.26 | 13 | 70.74 | mitochondrion | translational elongation | 6.82 |
| 16 | ATP synthase subunit beta, mitochondrial | ATP5B | P06576 | 56560.2 | 5.26 | 36 | 220.67 | mitochondrion | angiogenesis | 6.82 |
| 17 | 14-3-3 protein sigma | SFN | P31947 | 27774.2 | 4.68 | 12 | 49.46 | cytoplasm, nucleus | apoptosis | 6.07 |
| 18 | Protein SET | SET | Q01105 | 33489.0 | 4.23 | 23 | 85.7 | ER, nucleus, cytoplasm | negative regulation of neuron apoptosis | 5.93 |
| 19 | Glyceraldehyde-3-phosphate dehydrogenase | GAPDH | P04406 | 36053.4 | 8.57 | 35 | 225.36 | membrane, cytoplasm | glycolysis, neuron apoptosis | 5.76 |
| 20 | L-lactate dehydrogenase B chain | LDHB | P07195 | 36638.7 | 5.71 | 25 | 130.19 | cytoplasm | glycolysis, pyruvate metabolic process | 5.58 |
| 21 | 14-3-3 protein theta | YWHAQ | P27348 | 27764.4 | 4.68 | 13 | 47.11 | cytoplasm | regulation of progression through cell cycle | 5.54 |
| 22 | Phosphoglycerate kinase 1 | PGK1 | P00558 | 44615.0 | 8.30 | 38 | 209.46 | cytoplasm | gluconeogenesis, glycolysis | 5.26 |
| 23 | Protein disulfide-isomerase A6 | PDIA6 | Q15084 | 48121.6 | 4.95 | 21 | 110.7 | ER | cell redox homeostasis | 4.99 |
| 24 | Calreticulin | CALR | P27797 | 48141.8 | 4.29 | 22 | 121.57 | ER | cell cycle arrest, regulation of apoptosis | 4.86 |
| 25 | Keratin, type I cytoskeletal 18 | KRT18 | P05783 | 48058.0 | 5.34 | 36 | 257.94 | intermediate filament ,cytoplasm | cell cycle, regulation of apoptosis | 4.68 |
| 26 | Non-POU domain-containing octamer-binding protein | NONO | Q15233 | 54231.9 | 9.01 | 6 | 31.76 | nucleus | DNA repair, regulation of transcription | 4.12 |
| 27 | Vimentin | VIM | P08670 | 53651.9 | 5.06 | 33 | 285.76 | cytosol, intermediate filament | involved in apoptosis, cell motility | 4.11 |
| 28 | Alpha-enolase | ENO1 | P06733 | 47169.2 | 7.01 | 55 | 372.90 | plasma membrane nucleus | gluconeogenesis | 3.69 |
| 29 | 40S ribosomal protein SA | RPSA | P08865 | 32854.2 | 4.79 | 11 | 64.72 | plasma membrane, nucleus | signal transduction | 3.64 |
| 30 | 14-3-3 protein gamma | YWHAG | P61981 | 28302.7 | 4.80 | 23 | 94.06 | cytoplasm | G2/M transition of mitotic cell cycle | 3.45 |
| 31 | Tubulin beta-2B chain | TUBB2B | Q9BVA1 | 49953.4 | 4.78 | 36 | 262.69 | cytoplasm, microtubule | microtubule-based movement mitosis | 3.30 |
| 32 | Tubulin beta-3 chain | TUBB3 | Q13509 | 50433.0 | 4.83 | 27 | 197.23 | cytoplasm, microtubule | microtubule-based movement mitosis | 3.26 |
| 33 | Eukaryotic initiation factor 4A-I | EIF4A1 | P60842 | 46154.2 | 5.32 | 14 | 80.05 | cytosol | eIF4F complex translation | 3.15 |
| 34 | Keratin, type I cytoskeletal 19 | KRT19 | P08727 | 44092.2 | 5.04 | 13 | 88.72 | intermediate filament | response to estrogen stimulus | 3.11 |
| 35 | Tubulin beta-2C chain | TUBB2C | P68371 | 49831.3 | 4.79 | 50 | 332.39 | cytosol, microtubule | G2/M transition of mitotic cell cycle | 3.09 |
| 36 | Tubulin beta chain | TUBB | P07437 | 49671.1 | 4.78 | 44 | 307.32 | cytosol, microtubule | G2/M transition of mitotic cell cycle | 3.07 |
| 37 | Gamma-enolase | ENO2 | P09104 | 47268.8 | 4.91 | 7 | 40.62 | plasma membrane | gluconeogenesis, glycolysis | 2.87 |
| 38 | Polypyrimidine tract-binding protein 1 | PTBP1 | P26599 | 57221.6 | 9.22 | 15 | 67.87 | nucleoplasm, nucleolus | mRNA processing, RNA splicing | 2.74 |
| 39 | Heterogeneous nuclear ribonucleoprotein H3 | HNRNPH3 | P31942 | 36926.7 | 6.37 | 11 | 47.84 | nucleus, nuclear | mRNA splicing | 2.73 |
| 40 | Actin, cytoplasmic 2 | ACTG1 | P63261 | 41793.1 | 5.31 | 51 | 293.06 | cytoskeleton, cytosol | cell motility response to calcium ion | 2.72 |
| 41 | Heterogeneous nuclear ribonucleoprotein H | HNRNPH1 | P31943 | 49229.7 | 5.89 | 9 | 56.14 | actin cytoskeleton, cytoplasm | regulation of RNA splicing | 2.72 |
| 42 | Heterogeneous nuclear ribonucleoprotein R | HNRNPR | O43390 | 70943.4 | 8.23 | 7 | 54.9 | nucleus, cytoplasm | mRNA processing, RNA splicing | 2.55 |
| 43 | Keratin, type I cytoskeletal 17 | KRT17 | Q04695 | 48105.9 | 4.97 | 35 | 227.92 | cytoplasm intermediate | epidermis development | 2.54 |
| 44 | Actin, alpha skeletal muscle | ACTA1 | P68133 | 42051.3 | 5.23 | 31 | 197.80 | actin filament, cytosol | cell motility, response to calcium ion | 2.53 |
| 45 | Keratin, type II cytoskeletal 8 | KRT8 | P05787 | 53704.5 | 5.52 | 41 | 332.32 | cytoplasm, nucleoplasm | keratin filament cytoskeleton organization | 2.49 |
| 46 | ATP synthase subunit alpha, mitochondrial | ATP5A1 | P25705 | 59750.9 | 9.16 | 30 | 193.78 | mitochondrial, plasma membrane | negative regulation of cell proliferation | 2.48 |
| 47 | L-lactate dehydrogenase A chain | LDHA | P00338 | 36688.9 | 8.44 | 34 | 187.99 | cytosol | glycolysis, metabolic process | 2.46 |
| 48 | Prohibitin | PHB | P35232 | 29804.2 | 5.57 | 30 | 124.16 | mitochondrial membrane | regulation of apoptosis and cell growth | 2.39 |
| 49 | Heterogeneous nuclear ribonucleoprotein Q | SYNCRIP | O60506 | 69603.0 | 8.68 | 5 | 49.2 | mitochondrial membrane | mRNA processing, RNA splicing | 2.33 |
| 50 | Keratin, type II cytoskeletal 7 | KRT7 | P08729 | 51418.0 | 5.50 | 27 | 231.06 | Golgi apparatus, nucleus | DNA replication | 2.16 |
| 51 | Tubulin alpha-4A chain | TUBA4A | P68366 | 49924.7 | 4.95 | 28 | 169.99 | cytosol, microtubule | G2/M transition of mitotic cell cycle | 2.14 |
| 52 | Talin-1 | TLN1 | Q9Y490 | 269768.5 | 5.78 | 0 | 23.78 | [actin cytoskeleton](http://www.ebi.ac.uk/ego/DisplayGoTerm?id=GO:0015629), [cytosol](http://www.ebi.ac.uk/ego/DisplayGoTerm?id=GO:0005829) | cell adhesion, movement | 2.01 |
| 53 | Glutathione S-transferase Mu 3 | GSTM3 | P21266 | 26559.8 | 5.37 | 18 | 60.54 | cytoplasm | metabolic process | 2.00 |
| 54 | Cofilin-1 | CFL1 | P23528 | 18502.6 | 8.22 | 43 | 121.24 | cell membrane, cytoplasm | actin cytoskeleton organization | 2.00 |

*Cov. represents coverage; †S.U. represents scores unique; ‡FC represents fold change

**Table S2** Identification of down-regulated proteins in 6-shogaol treated HeLa cells using LC Chip Q-TOF MS/MS.

| No. | protein name | gene name | Swiss  Prot | protein  MW | protein  pI | Cov.* | S.U.^†^ | cellular component | biological process | FC^‡^ |
| --- | --- | --- | --- | --- | --- | --- | --- | --- | --- | --- |
| 1 | Clathrin heavy chain 1 structural molecule activity | CLTC | Q00610 | 191615.7 | 5.48 | 19 | 389.54 | membrane mitochondrion | mitosis, post-Golgi vesicle-mediated transport | -43.478 |
| 2 | Threonyl-tRNA synthetase, cytoplasmic | TARS | P26639 | 83435.6 | 6.24 | 17 | 170.9 | cytoplasm | threonyl-tRNA aminoacylation | -37.037 |
| 3 | Myosin-9 microfilament motor activity | MYH9 | P35579 | 226533.4 | 5.50 | 13 | 282.39 | cytoplasm | angiogenesis, cell-cell adhesion | -9.009 |
| 4 | Nucleoside diphosphate kinase B | NME2 | P22392 | 17298.1 | 8.52 | 56 | 125.90 | cytosol, nucleus | negative regulation of apoptosis | -7.299 |
| 5 | Fatty acid synthase process；acyl carrier activity | FASN | P49327 | 273401.0 | 5.99 | 13 | 330.99 | Golgi apparatus | fatty acid biosynthetic process | -5.208 |
| 6 | Plectin | PLEC | Q15149 | 531735.2 | 5.73 | 3 | 156.63 | cytosol focal adhesion | apoptosis | -4.386 |
| 7 | 40S ribosomal protein S19 | RPS19 | P39019 | 16060.6 | 10.31 | 29 | 61.09 | cytosolic, nucleolus | positive regulation of cell motility | -4.329 |
| 8 | Nucleoside diphosphate kinase A | NME1 | P15531 | 17148.8 | 5.83 | 48 | 111.92 | cytosol nucleus | regulation of apoptosis | -4.237 |
| 9 | Transferrin receptor protein 1 | TFRC | P02786 | 84871.8 | 6.18 | 10 | 95.09 | membrane | Proteolysis, transmembrane transport | -3.861 |
| 10 | Nuclear autoantigenic sperm protein | NASP | P49321 | 85238.1 | 4.26 | 5 | 37.95 | nucleus - cytoplasm | cell cycle, cell proliferation | -3.831 |
| 11 | 4F2 cell-surface antigen heavy chain | SLC3A2 | P08195 | 57945.2 | 5.20 | 23 | 133.59 | membrane melanosome | cell growth - calcium ion transport | -3.759 |
| 12 | Brain acid soluble protein 1 | BASP1 | P80723 | 22693.5 | 4.64 | 35 | 64.00 | plasma membrane | cell differentiation | -3.759 |
| 13 | Transketolase | TKT | P29401 | 67878.0 | 7.58 | 36 | 239.51 | cytosol | energy reserve metabolic process | -3.636 |
| 14 | Heterogeneous nuclear ribonucleoprotein U | HNRNPU | Q00839 | 90513.8 | 5.76 | 11 | 146.62 | cell surface nucleoplasm | cell killing, mRNA processing | -3.378 |
| 15 | Calnexin | CANX | P27824 | 67568.6 | 4.47 | 12 | 92.91 | endoplasmic reticulum | angiogenesis, protein secretion | -3.125 |
| 16 | Importin subunit beta-1 | KPNB1 | Q14974 | 97170.8 | 4.68 | 6 | 56.61 | nucleus - cytoplasm | protein import into nucleus, translocation | -2.703 |
| 17 | Phosphatidylethanolamine-binding protein 1 | PEBP1 | P30086 | 21056.9 | 7.01 | 26 | 59.2 | cytoplasm | none | -2.188 |
| 18 | ATP-dependent RNA helicase DDX3X | DDX3X | O00571 | 73243.8 | 6.73 | 11 | 82.37 | cytoplasm nuclear | speck interspecies interaction between organisms | -2.123 |
| 19 | Heat shock protein HSP 90-beta binding | HSP90AB1 | P08238 | 83264.6 | 4.97 | 38 | 431.69 | cytosol melanosome | positive regulation of nitric oxide biosynthetic process | -2.123 |
| 20 | T-complex protein 1 subunit theta | CCT8 | P50990 | 59620.9 | 5.42 | 11 | 69.43 | cytosol | 'de novo' posttranslational protein folding | -2.092 |
| 21 | X-ray repair cross-complementing binding protein 5 | XRCC5 | P13010 | 82705.0 | 5.55 | 8 | 76.12 | cytoplasm nucleus | negative regulation of transcription | -2.049 |
| 22 | Protein canopy homolog 2 | CNPY2 | Q9Y2B0 | 20652.3 | 4.81 | 23 | 46.84 | endoplasmic reticulum | none | -2.008 |

*Cov. represents coverage; †S.U. represents scores unique; ‡FC represents fold change
